# Teachers' autonomy support and student engagement: A systematic literature review of longitudinal studies

**DOI:** 10.3389/fpsyg.2022.925955

**Published:** 2022-08-22

**Authors:** Dong Yang, Peng Chen, Huanhuan Wang, Kai Wang, Ronghuai Huang

**Affiliations:** ^1^Smart Learning Institute, Beijing Normal University, Beijing, China; ^2^College of Education, Capital Normal University, Beijing, China; ^3^Center for Teacher Education Research, Beijing Normal University, Beijing, China

**Keywords:** teaching practice, autonomy support, student engagement, literature review, longitudinal studies

## Abstract

Autonomy support is one of the most crucial determinants of teaching practice for student engagement. No literature review on the relations between autonomy support and student engagement existed to the best of our knowledge. Therefore, this study presents a systematic literature review from perspectives of landscapes, methodology characters, patterns of identified studies, and autonomy-supportive strategies. Overall, 31 articles were reviewed. Followed by PRISMA guidelines, the results yielded several interesting facts: First, studies on such topics surged starting from 2015 and were mostly conducted in the United States (32%) and Korea (16%). Publications were scattered but heavily gathered around psychological and educational journals such as the *Journal of Educational Psychology* (9.7%)*; Learning and Instruction (9.7%)*. Most often, studies recruited participants from upper secondary schools (58%). Data were collected using solely questionnaires (93.5%) following a two-wave design (51.6%) and were analyzed by applying structural equation models (48.4%). Moreover, most of the studies failed to provide concrete autonomy-supportive teaching strategies. Instead, quite often studies (93%) investigated its relations with student engagement from a macro perspective. Within mentioned strategies, they were mostly related to the teaching process, there is a limited investigation of autonomy-supportive teaching practice used before and after instruction. This pattern of results suggested an urgent need for more longitudinal studies on specific teaching strategies that hold the potential to maximize student engagement. Limitations and suggestions for future studies were provided accordingly.

## Introduction

Research on student engagement has gained increasing popularity recently as it holds the potential to address problems such as early dropout and poor achievement. The concept of engagement is appealing as it is malleable and sensitive to changes in both teachers' practices (Fredricks et al., 2016). Therefore, engagement was used as a key target for interventions and as an explicit goal of many school improvement programs (Appleton et al., 2008). Recent studies also emphasized the importance of autonomy-supportive teaching practice on student engagement, including teachers' dialogic discourse practice (Böheim et al., 2021) and classroom structure (Cheon et al., 2020). Teacher's autonomy support refers to the degree of latitude teachers give their students during learning activities (Reeve, 2009), including teaching behaviors that detect and nurture kids' needs, interests, and preferences, as well as providing chances in the classroom for students to use their motivations to direct their learning and activities (Reeve et al., 2004). There is evidence that when teachers learn to provide autonomy support in the classroom, it benefits both teachers (e.g., teaching efficacy, teaching skill, and teaching wellbeing) (Rimm-Kaufman and Sawyer, 2004; Cheon et al., 2014) and students (e.g., motivations, classroom engagement, and skill development) (Cheon et al., 2020). More recently, literature review focused on studies applying autonomy-supportive teaching interventions has found that autonomy-supportive teaching is malleable as it can be gained during instruction (Reeve and Cheon, 2021). Participants in most of the intervention studies manifested effective teaching behaviors such as avoiding uttering solutions/answers, being responsive to student-generated questions, spending more time listening, and providing a meaningful rationale (McLachlan and Hagger, 2010; Reeve and Cheon, 2021). Those aforementioned autonomy-supportive teaching behaviors, once learned during the teaching practices, endured (Cheon and Reeve, 2013; Tilga et al., 2020).

One critical factor for boosting student engagement is teaching/motivational styles (i.e., autonomy support from teachers). Teachers who are autonomy-supportive help their students to develop internal motivational resources that promote their engagement in learning (Connell and Wellborn, 1991; Reeve, 2009). Moreover, they present students with meaningful choices between tasks or activities, explain why classroom activities matter, and allow them to pursue their own goals and make decisions on their actions (Reeve et al., 2020).

Although a large body of research has been conducted on the relations between autonomy support and learning engagement, there is still a dearth of studies that synthesize the previous works on such topics. Moreover, recent reviews on student engagement emphasized heavily the technology-mediated environment(e.g., Henrie et al., 2015; Schindler et al., 2017), and it seems that review work on autonomy support is frequently seen in autonomy support in the workplace (e.g., Slemp et al., 2018), and in the field of sports and exercise (e.g., Pérez-González et al., 2019; Raabe et al., 2019). There was one review of the effect of teaching practice on student engagement (i.e., Harbour et al., 2015), that summarized good teaching practices that hold promise to boost student engagement. This particular review, however, failed to provide an intensive picture of how autonomy support could contribute to engagement. A systematic literature review on such a topic provides at least two benefits: first, according to a recent report, the impact of the pandemic on education will last longer than we expected (Dorn et al., 2020), and the damage of the pandemic to individuals goes from learning loss to even loss of earning in students' future working-life (Dorn et al., 2020). Keep students motivated in learning is important during emergency remote teaching, by enhancing autonomy support, teachers can play a role in promoting student engagement; Second, it may provide both researchers and teachers/instructors insights on how to keep students positively engaged in schoolwork, especially in the new teaching normal such as emergency remote teaching (ERT) due to pandemic. In this review, we aim to provide a systematic review of autonomy support and student engagement from four perspectives, namely the landscape of studies, methodologies characters, patterns, and the proposed teachers' autonomy support strategies.

## Methods

We conducted a systematic literature review of the published literature on empirical longitudinal studies of autonomy support and student engagement in the past 20 years. A systematic review was chosen because it provides summaries of the state of knowledge in a field from which future research goals may be established, answers issues that individual studies could not; highlights main research flaws that should be addressed in future studies (Page et al., 2021). Therefore, we opt for a systematic review approach to understanding how the studies on teacher autonomy support and student engagement were conducted. To do this, we followed the Preferred Reporting Items for Systematic reviews and Meta-Analyses (PRISMA; Moher et al., 2009) framework when conducting our scoping review. This review covered four topics: (1) the landscape of studies, (2) methodological issues, (3) patterns of previous studies, and (4) the strategies & effectiveness of teachers' autonomy support on student engagement.

### Searching strategy

The literature search was performed within databases such as ISI Web of Knowledge, Science Direct, Scopus, and Google Scholar. Those databases were chosen for their breadth in education, psychology, and technology. We included peer-reviewed journal articles published from January 2000 to March 2022. Three key search terms used on the databases were: “autonomy support” “student engagement” and “longitudinal.” Although Similar terms such as “involvement” and “participation” can be found in the literature, we chose to focus only on articles using the word “engagement” in the abstract section, expecting that it would have direct connections with student engagement. We used alternative terms in the searching strings regarding engagement to expand the results, as described in Table 1.

**Table 1:** Search terms and strings.

**Items**	**Search terms**	**Boolean**
Autonomy support	“Autonomy-supportive environment” OR “autonomy-supportive interventions” OR “autonomy-supportive teaching” OR “motivational styles” OR “support for autonomy” OR “dialogic discourse practice” OR “supportive instruction practices” OR “classroom climate” OR “teacher support”	AND
Student engagement	“School engagement” OR “engagement in school” OR “student engagement” OR “pupil engagement” OR “learner engagement” OR “emotional engagement” OR “cognitive engagement” OR “behavioral engagement” OR “agentic engagement” OR “academic engagement”	AND
Longitudinal study	“Longitudinal” OR “longitudinal design” OR “longitudinal study” “longitudinal sample” OR “longitudinal associations” OR “longitudinal increase” OR “longitudinal survey” OR “panel study”	

### Inclusion and exclusion criteria

To ensure a quality collection of literature, we only chose peer-reviewed journal articles published in English. Since one objective of this study is to explore the topics of teachers' autonomy support and student engagement, we only selected the empirical studies with a longitudinal design. We only chose longitudinal studies as its principal advantage to understand intraindividual change compared with the cross-sectional studies which mainly focused on interindividual differences (Schaie and Hofer, 2001). Detailed inclusion criteria were shown in Table 2.

**Table 2:** Inclusion and exclusion criteria.

**Inclusion criteria**	**Exclusion criteria**
Journal articles	Short reports, conference papers, book chapters, etc.
Peer-reviewed	Not peer-reviewed
Empirical studies	Non-empirical studies and theoretical studies
Written in english	Written in other languages
Longitudinal studies	Non-longitudinal studies
Published between 2020 and 2022	Published before 2000 or after the time of writing
Focused on teachers' autonomy support & student engagement	Focused on parents' autonomy support, work engagement, teacher engagement, etc.

### Screening process

**Figure 1:**
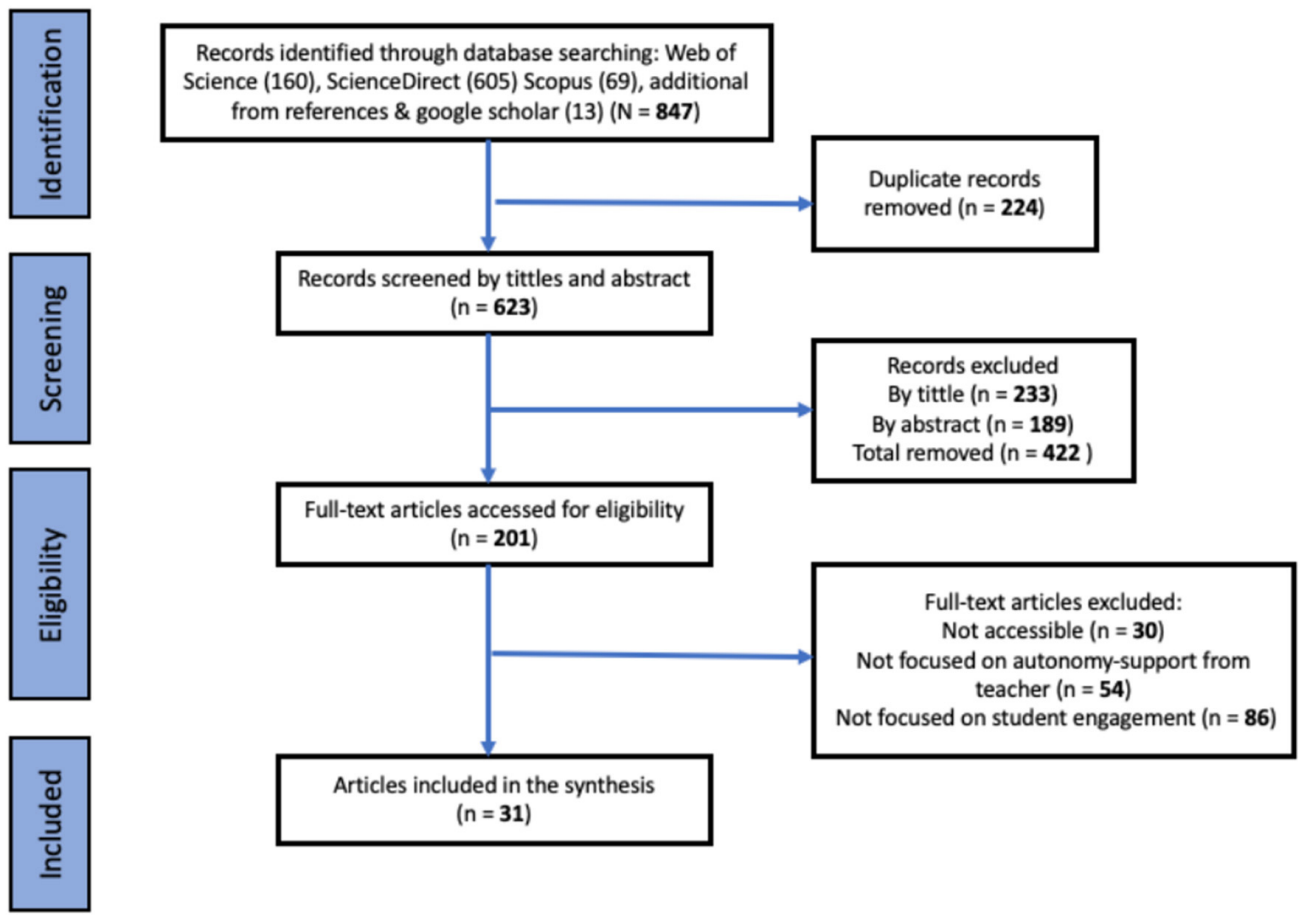
PRISMA style article identification flow.

A comprehensive search across databases such as Web of Science, Scopus, ScienceDirect, and additional sources from Google Scholar resulted in 847 articles, and screening of the title and abstracts (*N* = 623) articles resulted in empirical articles that met inclusion criteria. Then we carefully went through each article applying the inclusion and exclusion criteria (see Figure 1), this process yielded a total of 31 articles for final synthesis. The detailed identification flow is shown he Figure 1.

## Findings

### What are the landscapes of the identified studies?

#### Countries

We identified countries by the affiliation of the first author. Overall, approximately one out of three studies were conducted in the United States (*N* = 10; 32%); Korea ranks second with an output of five articles (16%). In addition to States and Korea, China and Germany both contributed three articles (*N* = 6; 19%), while the Netherlands added two studies (6%) to the pool. The rest of the studies (*N* =6 ; 19%) scattered across Canada, Israel, Peru, Portugal, Spain, and Turkey. No article on such a topic was identified across African countries. See Table 3 for more details.

**Table 3:** A summary of countries and participants of identified studies (*N* = 31).

**Country/ place of study**	**N**	**Article**	**No. of participants: student (teachers)**
Canada	1	1. Archambault et al. (2020)	696 (67)
China	3	1. Wei et al. (2020)	1624 (–)
		2. Yu et al. (2015)	356 (–)
		3. Yu et al. (2016)	236 (–)
Germany	3	1. Böheim et al. (2021)	450 (19)
		2. C. Frommelt et al. (2021)	751 (–)
		3. Lazarides and Rubach (2017)	751 (–)
Israel	1	1. Kaplan (2018)	144 (–)
Japan	2	1. Jiang and Tanaka (2022)	199 (87)
		2. Oga-Baldwin and Nakata (2015)	344 (–)
Korea	5	1. Cheon et al. (2016)	1,017 (19)
		2. Cheon et al. (2020)	4,195 (81)
		3. Jang et al. (2012)	500 (–)
		4. Jang et al. (2016)	366 (–)
		5. Reeve et al. (2020)	1,422 (22)
Netherland	2	1. Flunger et al. (2022)	202 (12)
		2. Zee and Koomen (2020)	472 (63)
Peru	1	1. Matos et al. (2018)	336 (–)
Portugal	1	1. Moreira and Lee (2020)	2,676 (–)
Spain	1	1. Núñez and León (2019)	448 (–)
Turkey	1	1. Michou et al. (2021)	257 (–)
United States	10	1. Baker et al. (2017)	120 (6)
		2. Kiefer and Pennington (2017)	209 (–)
		3. Mustafaa et al. (2017)	571 (31)
		4. Patall et al. (2019)	208 (41)
		5. Patall et al. (2018b)	208 (41)
		6. Patall et al. (2018a)	208 (41)
		7. Reeve et al., 2004	–(20)
		8. Ruzek and Schenke (2019)	910 (–)
		9. van Ryzin et al. (2009)	283 (–)
		10. Williams et al. (2018)	113 (3)

“– ” means data not reported. One study used only teacher samples (Reeve et al., 2004), others nearly half of the studies (N = 14; 45%) used collected data from the perspectives of both students and teachers.

**Figure 2:**
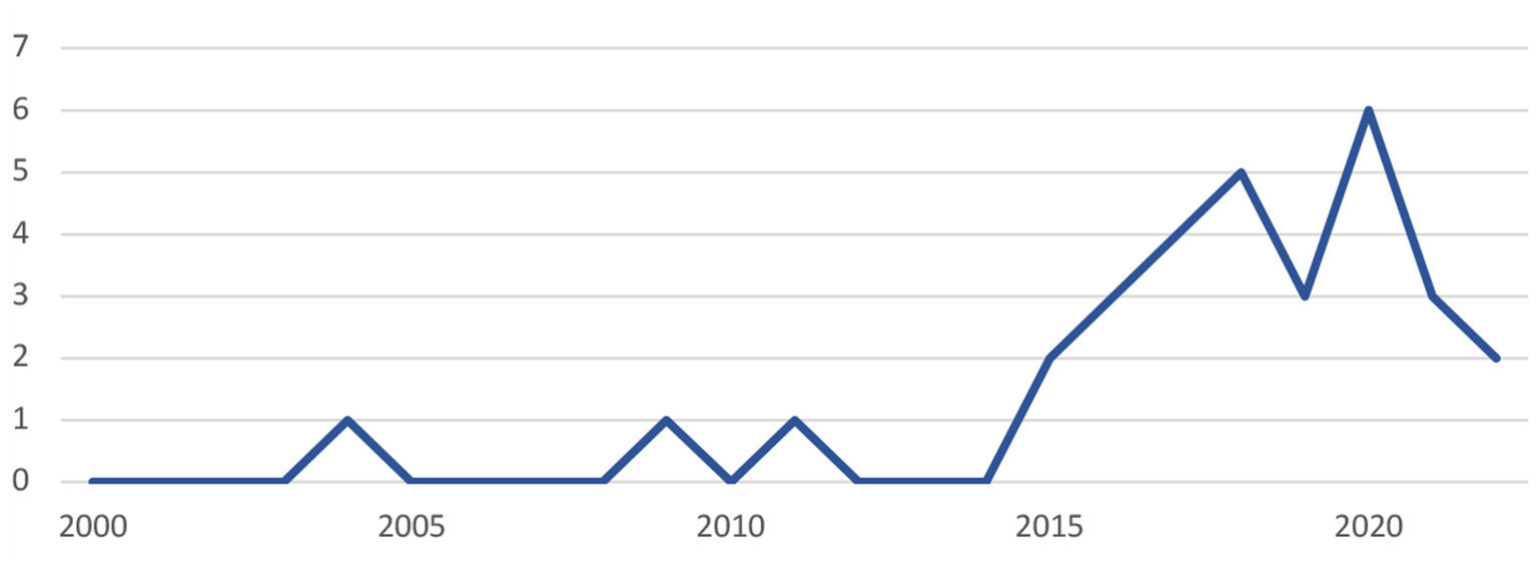
Number of publications by year (Till 20 March).

**Figure 3:**
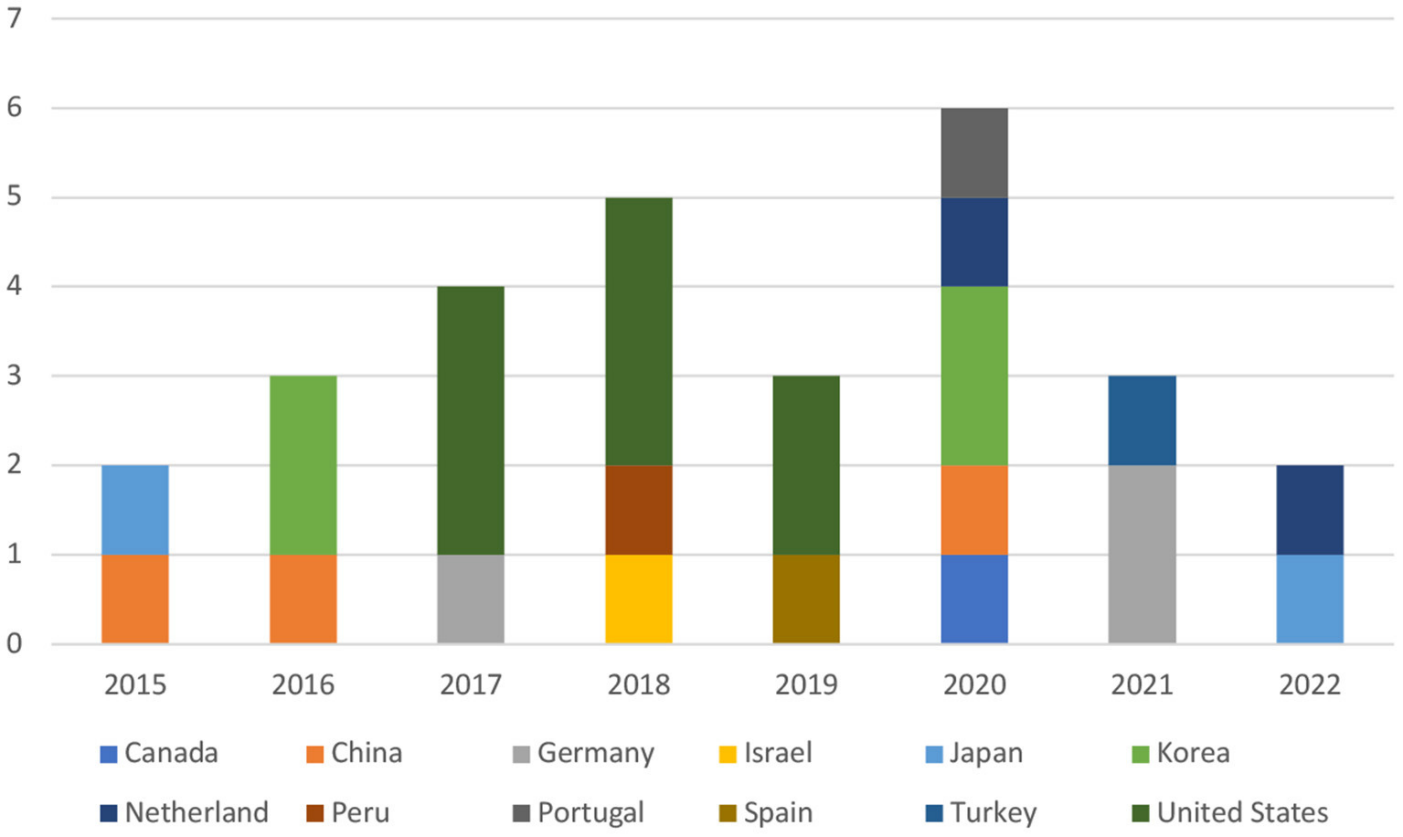
Timeline of publication by the geographic area between 2015 and 2022.

As indicated in Figure 2, at a first sight, the studies on autonomy support and student engagement seem to be scarce in the first decade, with only three articles (10%) screened before the year 2015. Then starting from 2015, academic output on the topic is gaining momentum till the year 2021, which contributed 90% of the total number. Indicating that the topic is getting increasing attention during the past decade. To make the trend clearer, we made a histogram that vividly shows the trend from 2015 to 2022 March in each country. See Figure 3 below.

#### Journals

In terms of publication, publications are mainly located in Psychological and Educational journals. *Journal of Educational Psychology* (9.7%)*; Learning and Instruction* (9.7%) lead the publications, representing three articles separately. Besides, journals such as *Teachers and Teaching: Theory and Practice; Middle Grades Research Journal both* contributed two articles, accounting for 13% of the total sum. The rest of the articles were scattered across different journals, mainly in psychology journals such as *Journal of Adolescence*; *Journal of Youth and Adolescence*. Several publications were identified in the Sports and Science related journals, for example, in *Science Education, Journal of Sport and Exercise Psychology*, etc. Refer the Table 4 below and Table 3 above for full details.

**Table 4:** Publications of identified articles.

**Journals**	**N of study**	**Percentage**
Contemporary Educational Psychology	1	3.2%
Cyberpsychology, Behavior, and Social Networking	1	3.2%
Educational Psychology	1	3.2%
European Journal of Psychology of Education	1	3.2%
Interdisciplinary Education and Psychology	1	3.2%
International Journal of Behavioral Development	1	3.2%
International Journal of STEM Education	1	3.2%
Japanese Psychological Research	1	3.2%
Journal of Adolescence	1	3.2%
Journal of Applied Developmental Psychology	1	3.2%
Journal of Educational Psychology	3	9.7%
Journal of Experimental Education	1	3.2%
Journal of Sport and Exercise Psychology	1	3.2%
Journal of Research in Childhood Education	1	3.2%
Journal of Youth and Adolescence	1	3.2%
Learning and Individual Differences	1	3.2%
Learning and Instruction	3	9.7%
Learning, Culture, and Social Interaction	1	3.2%
Mathematics Education Research Journal	1	3.2%
Middle Grades Research Journal	2	6.5%
Motivation and Emotion	1	3.2%
Science Education	1	3.2%
Social Psychology of Education	1	3.2%
Teaching and Teacher Education	1	3.2%
Teachers and Teaching: Theory and Practice	2	6.5%

#### Participants and samples

Identified studies ranged from a small sample size of 20 (Reeve et al., 2004) to large size of 4,195 (Cheon et al., 2020), M_sample_ = 671. Fourteen studies (45%) reported both samples from students and teachers, while 16 (52%) studies presented only the data on student participants. One study only used teacher samples (Reeve et al., 2004). The mean age of students is 15.44, mean age for teachers is 37.14 with an average teaching experience of 11.5 years (based on information available). In terms of educational level, more than 90% (*N* =2 8) of the identified studies were located in the K12 context. Among them, a large body of studies (*N* = 58%) focused on grade 7–12 level, nearly one out of five (*N* = 6, 19.4%) concerned lower grades 1–6, and four studies (12.9%) used both samples from K6 and K7 to K12 levels. Only three studies focused on the undergraduate groups, representing 9.7% of the total sum. Refer to Table 5 for more info.

**Table 5:** The level of students studied.

**Level of education**	**Frequency**	**Percent (%)**	**Studies (examples)**
K6	6	19.4%	Baker et al., 2017; Mustafaa et al., 2017
K7–12*	18	58.0%	Patall et al., 2019; Reeve et al., 2020
K6 & K7–12	4	12.9%	Ruzek and Schenke, 2019; Michou et al., 2021
Undergraduates	3	9.7%	Matos et al., 2018; Jiang and Tanaka, 2022
Total	31	100%	–

(1) ^*^ studies reported sample as “middle/high school” were cataloged as K7–K12 level, as corresponded to secondary school.

### What are the features of methodology in current studies?

#### Study design

In features of methodology, we mainly looked at the study design (waves and period), data type, analysis techniques, theories, and instruments that were utilized in identified studies. First, we found approximately half of the studies (*N* = 16; 51.6%) used a two-wave longitudinal design, one out of four studies (*N* = 8; 25.8%) feature a three-wave design, while four studies applied a four-point measurement (9.7%). Meanwhile, the majority of the studies (*N* = 19; 61.3%) followed a middle-term timespan (that last from several months to a year); seven (22.7%) studies reported a short-term data collection schedule, four (9.7%) with long-term timespan, while only one study features a continuous measurement as described in Table 6.

**Table 6:** Characteristics of study design (waves of data and period).

**Waves/types of study**	**N**	**Percentage**
Interval	4	12.9%
1 interval	16	51.6%
2 intervals	8	25.8%
3 intervals	3	9.7%
Total	31	100%
Continuous	1	3.3%
Short-term	7	22.7%
Middle-term	19	61.3%
Long-term	4	9.7%
Total	31	100%

Interval, waves of measurement unclear; continuous, constant measurement across time; momentary, measured across seconds or minutes; Short-term, measured across days or weeks; middle-term, measured across months to one year; long-term, measured across more than one year.

#### Data and analysis plan

Second, on data type and analysis plan, studies mostly (*N* = 29, 93.5%) relied on a self-reported questionnaire to capture data, making observation data less appealing comparatively (*N* = 2, 6.5%). Statistically speaking, the studies seem to be obsessed with structural equation models (SEM; *N* = 15, 48.4%), and using multilevel regression analyses such as hierarchical linear modeling (HLM) approaches (*N* = 7; 22.7%). This result is not surprising as a study with repeated measurements usually resulted in a nested data structure (Goldstein et al., 1993). Other analysis techniques, though less favored, were path analysis, HMRA, and repeated measures. Out of the total of 31 articles, 26 (83.9%) reported using an intervention/experimental design. See Table 7 below.

**Table 7:** Data type and analysis techniques.

**Data type**	**N**	**Percentage**
Questionnaire data	29	93.5%
Observation data	2	6.5%
Total	31	100%
**Analysis techniques**		
HLM	7	22.7%
HMRA	2	6.4%
SEM	15	48.4%
Path analysis	1	3.2%
Repeated measures	2	6.4%
Others	4	12.9%
Total	31	100%
**Intervention**		
Yes	5	16.1%
No	26	83.9%
Total	31	100%

#### Theoretical issues

As expected, more than two out of three (67.7%) studies applied self-determination theory (SDT) as the grounding theory (e.g., Lazarides and Rubach, 2017; Michou et al., 2021; Jiang and Tanaka, 2022), due to its argument that students' motivation and engagement in the classroom are influenced by how they perceive their learning environment and how teachers meet their basic psychological needs (Ryan and Deci, 2000). Besides SDT, three (around 10%) studies referred to social-cognitive theories (Ruzek and Schenke, 2019) or stage–environment fit theory (Yu et al., 2015, 2016) to underpin their studies. In addition, seven articles were unclear on the underpinning theories (e.g., Kiefer and Pennington, 2017; Frommelt et al., 2021). See Table 8 below.

**Table 8:** Theories used in identified studies.

**Theory**	**N of study**	**Percentage**
Self-determination theory	21	67.7%
Social-cognitive theories	1	3.2%
Stage–environment fit theory	2	6.5%
Others (theory not clear)	7	22.6%

The most frequently investigated aspect of student engagement was behavioral engagement (*N* = 23, 74.2%), followed by cognitive and emotional engagement (54.8% and 58.1% separately). Almost one out of three studies worked on the agentic perspective, representing 35.5% of the total. Among them, nine (29%) articles measured student engagement from agentic, behavioral, cognitive, and emotional dimensions (e.g., , Cheon et al., 2016; Matos et al., 2018; Núñez and León, 2019), and five studies researched student engagement from the popular “BCE” perspective (i.e., Yu et al., 2015, 2016; Mustafaa et al., 2017; Archambault et al., 2020; Wei et al., 2020); while four studies concerned only on behavioral and emotional aspects (e.g., Reeve et al., 2004; van Ryzin et al., 2009). In addition, four (12.9%) articles were concerned with less frequently used dimensions such as social engagement (e.g., Baker et al., 2017). Details were presented in Table 9 below.

**Table 9:** Dimensions of student engagement studies concerned.

**Dimensions**	**Frequency**	**Percentage**
Agentic engagement	11	35.5%
Behavioral engagement	23	74.2%
Cognitive engagement	17	54.8%
Emotional engagement	18	58.1%
Others	4	12.9%

In addition, popular instruments used in studies were presented in Table 10. Due to the space limit, we do not cover this in detail.

**Table 10:** Most frequently used instruments.

**Dimensions of measurement**	**Names of instruments (authors)**	**Study examples**
Autonomy support	Learning climate questionnaire (LCQ; Williams and Deci, 1996) (*N* = 8)	Núñez and León, 2019; Cheon et al., 2020
**Engagement**		
Agentic engagement	Agentic engagement scale (Reeve, 2013) (*N* = 11)	Patall et al., 2018a; Reeve et al., 2020
Behavioral engagement	Engagement vs. disaffection with learning measure (Skinner et al., 2009) (*N* = 11)	Matos et al., 2018; Zee and Koomen, 2020
Cognitive engagement	Metacognitive strategies questionnaire (Wolters, 2004) (*N* = 6)	(Jang et al., 2012)
Emotional engagement	Engagement vs. disaffection with learning measure (Skinner et al., 2009) (*N* = 11)	Cheon et al., 2016; Patall et al., 2018b

Several studies used a self-developed questionnaire or coding frame (N = 6), for example, Oga-Baldwin and Nakata (2015) and Reeve et al. (2004) (coding frame), therefore they are not the scope of discussion of this part.

### Patterns of identified studies

In terms of the pattern of studies, most of the studies feature either bottom-up (i.e., autonomy support impacting student engagement) or top-down (student engagement impacting autonomy support) models that explain the bi-directional relationship between autonomy-supportive teaching strategies and student engagement. For example, a large body of studies (*N* = 20; 64.5%) utilized structural equation models or path analysis to understand the relations between teacher autonomy support and student engagement that we call the “TS” pattern. A significant amount from the rest of the studies (*N* = 7; 22.5%) added needs satisfaction into the equations, testing its mediating role in relations between autonomy support and student engagement, this was coded as the “TNS” pattern. Two studies concerned with how the engagement could contribute to teacher autonomy, and in turn, how the perceived autonomy support could boost further engagement. This was named as “ST” pattern. While a significant amount of the rest studies (*N* = 7; 22.5%) added needs satisfaction into the equations, testing its mediating role in relations between autonomy support and student engagement, this was coded as a “TNS” pattern. Unfortunately, two studies (6.5%) failed to indicate any similar pattern (as indicated in Table 11 below).

**Table 11:** Typical patterns of identified studies.

**Pattern**	**N of study**	**Percentage**
Teacher autonomy support –> student engagement (TS)	20	64.5%
Teacher autonomy support –> needs satisfaction–> student engagement (TNS)	7	22.5%
Student engagement –> teacher autonomy support (ST)	2	6.5%
Others (pattern unclear)	2	6.5%

### Which autonomy-supportive strategies were proposed?

Not all studies proposed concrete autonomy-supportive strategies. Still, from the texts, we can summarize several. Grounded in the early work of Reeve et al. (2004) and theories such as SDT (Ryan and Deci, 2000, 2002), most of the strategies used in screened articles include instructional behaviors such as taking the students' perspective (e.g., teaching students' preferred ways), invitational language, provide explanatory rationales, accept mistakes and negative affect, and display patience toward teaching and students (e.g., Jang et al., 2016; Reeve et al., 2020). In addition to autonomy-supportive teaching, a dialogic discourse that is structured, purposeful, interactive, and cumulative as well as guiding was also suggested, for the purpose to maximize student engagement (Böheim et al., 2021). Different from those aforementioned strategies that focused intensively on the teaching process (i.e., classroom teaching), Baker et al. (2017) investigated the effectiveness of teaching framing strategies (e.g., collaborative rule-setting, establishing procedures, or setting goals for interaction and expectations) that occurred before class time, one perspective that deserves more attention.

Most often, the proposed strategies were found effective to promote student engagement (e.g., Baker et al., 2017; Kiefer and Pennington, 2017). However, there are still controversial findings. For example, the study by Ruzek and Schenke (2019) concluded that students' perception of classroom autonomy support was unrelated to students' motivation and engagement among secondary school students, but students' behavioral engagement positively affected the bidirectional connections between their perceptions of autonomy support and academic stress. More details are provided below in Table 12. Due to space limitations, we present only examples here.

**Table 12:** Autonomy supportive strategies used to promote student engagement (examples).

**Authors**	**Strategies**	**Effectiveness**
Baker et al. (2017)	Teaching framing (i.e., collaborative rule-setting, establishing procedures, or setting goals for interaction and expectations)	Positive
Böheim et al. (2021)	Structured, purposeful, interactive, and cumulative as well as supportive and guiding dialogic discourse	Positive
Cheon et al. (2016); Jang et al. (2016), and Reeve et al. (2020)	Instructional behaviors including: take the students' perspective (e.g., teaching in students' preferred ways), invitational language, providing explanatory rationales, accepting negative effects, displaying patience	Positive
Oga-Baldwin and Nakata (2015), Lazarides and Rubach (2017), and Kiefer and Pennington (2017)	Provide choices, offer respect, show expectations, relevance	Positive
Ruzek and Schenke (2019)	Teachers seek students' perspectives, and respecting their opinions and have standards/expectations for student's efforts, and challenge students to go beyond what they know	Unrelated
van Ryzin et al. (2009)	Teacher-related belongingness (i.e., teacher support)	Positive

## Discussion

This review explored the basic pillars and landscapes of longitudinal studies on teachers' autonomy support and student engagement. Using a systematic literature review approach, and based on a literature pool of 31 articles (that featuring 20,804 participants), we found the available evidence as presented below:

First, we found that research on the topic mostly occurred in the United States and Korea, the rest of the studies scattered across several European and Asian countries, and there is an underrepresentation of African authors. Although we aimed to search literature from the past two decades, most of the identified studies were conducted from 2014 to 2015, especially on autonomy support and agentic engagement. This is understandable, as the concept of agentic engagement was originally proposed in the year 2013 by Reeve (2013) , thus it is not surprising that the research on such a theme surged since then. In terms of the sample, a large body of the studies recruited upper secondary school students as the samples, and almost every study was set in the classroom environment. This is probably because engagement has been regarded as a concept holding promise for improving reform and significant intervention targets particularly at the secondary level (Appleton et al., 2006; Fredricks et al., 2016). Simultaneously this means that there is a dearth of studies that focus on the underrepresented undergraduate group, and other learning environments such as blended learning and emergency remote teaching (ERT). Thus, we argue that there is a need to shift research focus on autonomy support and student engagement in the context of college teaching and in other new teaching normal such as ERT, a term/field that need consistent attention under the current situation, as students already experienced tremendous learning loss due to the pandemic (Dorn et al., 2020).

Secondly, on characters of methodology. Regards research design, most of the longitudinal studies applied two-wave design across a period of several months to a year. Meanwhile, almost all studies depended on a self-report survey (e.g., questionnaires) for data collection. While questionnaire data is the most common method for assessing student engagement and it is useful in collecting data on students' subjective perceptions, rather than just gathering objective data on behavioral markers such as attendance or assignment completion rates (Appleton et al., 2006). Some argue that questionnaires should only be harassed to access emotional and cognitive engagement which are not directly observable (Fredricks et al., 2016), thus other dimensions such as perceived autonomy support and behavioral engagement are observable sometimes. In addition to the questionnaire, provide observation, semi-structured interview, or even experience sampling methods (ESM; Larson and Csikszentmihalyi, 2014) that capture students' daily experience of teaching practice and engagement (e.g., Patall et al., 2018b), may add extra nuances to our understandings of complex interactions between autonomy support and student engagement. Across studies, the behavioral aspect of engagement was the most investigated (74.2%), probably because it is the only engagement dimension that contributed significantly to school dropout (Archambault et al., 2009), and can be manifested in observable activities.

Moreover, from the patterns of previous studies, we know that most of the studies failed to provide concrete autonomy-supportive teaching strategies, instead, quite often studies followed the schema such as exploring the “*teacher autonomy support –*> *needs satisfaction–*> *student engagement*” relations from a general-purpose. Research into how specific teachers' behavior can affect student engagement is becoming increasingly urgent, as the world (e.g., COVID-19 pandemic, district conflicts) and students nowadays are changing, both culturally and psychosocially. Educators need to search for effective ways to meet the challenges presented by the complex world, engage students in the new teaching normal (i.e., emergency remote teaching due to pandemics), and prevent dropouts.

In terms of autonomy support strategies, this review has found several distinct teaching strategies, and mostly they were grounded and underpinned by the early work of Ryan and Deci (2000, 2002) and Reeve et al. (2004). Meanwhile, a large body of aforementioned autonomy-supportive strategies was related to the teaching process, therefore investigation of autonomy-supportive teaching practice undertaken before and after the instruction process was insufficient to some extent. From the literature, one specific strategy proposed is to develop structured and interactive dialogic discourse between teachers and students (Böheim et al., 2021). Early research has repeatedly proven that the quality of a classroom discourse has an impact on students' learning behavior (Mercer and Dawes, 2014; Resnick et al., 2018), and it can be highly effective when students have the opportunity to discuss diverse ways of thinking, elaborating on their perspectives, and develop knowledge constructively and collaboratively (Michaels and O'Connor, 2015; Wilkinson et al., 2017). From this standpoint, further works on how dynamic classroom discourse could contribute to active learning and engagement are pertinent.

Last but not least, our review found that, in general, teachers' autonomy support hold promises to maximize student engagement. In educational practice, this means that teachers are suggested to use effective teaching strategies such as collaborative rule-setting, establishing procedures, or setting goals for interaction and expectations (Baker et al., 2017). When delivering courses, purposeful, interactive, and cumulative, as well as supportive dialogic discourse, is encouraged. Moreover, teachers should bear in mind that when they take the students' perspective (e.g., teaching in students' preferred ways), provide explanatory rationales, accept negative effects, and display patience, they are somehow proving an autonomy-supportive teaching environment. Meanwhile, schools should realize that teachers may have different teaching styles (be it autonomy-supportive or autonomy suppressing), thus carrying out customized teacher training programs is necessary.

## Limitations and future work

Several limitations existed in this review study. First, this study used only 31 articles based on inclusion criteria, and we only searched the term *engagement*, instead of using *involvement or participation*, to get additional results. However, it is understandable as the term engagement is more accurate and commonly used across studies (Fredricks et al., 2016). In this study, we only focused on longitudinal studies that feature several waves of data collection, which implies that the same participants are assessed repeatedly and thus other empirical studies were excluded for analysis. Simultaneously, the hierarchical/nested data structure may lead to measuring dependency and thus violates the assumptions underlying the general linear model (GLM; Schnettler et al., 2020). We believe that an independent study/review can compensate for this. In addition, from this view, we learned that a large body of research has focused on autonomy-supportive teaching as a foundation for student motivation and engagement. However, several recent studies have shown that a combination of two teaching styles, namely autonomy-support and structure, can be highly effective for student engagement (Vansteenkiste et al., 2012; Baker et al., 2017; Archambault et al., 2020). In line with this argument and potential constraints of this review, future work might test the effectiveness of structure (the volume and clarity of information provided to students about an activity, including the teacher's expectations concerning educational outcomes and how students are expected to achieve these outcomes, see Jang et al., 2010) with other teaching style factors, or conduct meta-analysis to explore how effective the teaching style (e.g., autonomy support, structure) is on student engagement. In our review, the behavioral aspect of engagement was mostly investigated (74.2%), probably because it is the only engagement dimension that contributed significantly to school dropout (Archambault et al., 2009). Therefore, further investigations are required to testify to the importance of other forms of engagement (e.g., agentic engagement) on student engagement across various learning environments.

## Conclusion

Longitudinal studies of teachers' autonomy support and student engagement were explored in a systematic literature review. The main concern of this review is to provide an in-depth review of landscapes, methodology used, trends/patterns of studies, and autonomy-supportive strategies. The main takeaway is that the studies on teachers' autonomy support and student engagement seem to concentrate in countries such as the United States and Korea, while largely underrepresented in African countries. Publications were scattered in the fields of psychology and education. Studies tend to follow a mid-term, two-wave data collection schema using self-reported questionnaires and analyzed by applying SEM. However, most of the studies failed to provide concrete autonomy-supportive teaching strategies, instead, they normally measured autonomy support and student engagement from a broad scale. As stated in the self-determination theory (Ryan and Deci, 2000), student's motivation and engagement in the classroom are influenced by how they perceive their learning environment and how teachers meet their basic psychological needs. In the face of everyday classroom challenges and at times of crisis, students need to display resilience by responding with increased engagement. Therefore, more in-depth exploration of the concrete teaching strategies that boost student engagement, thus preventing school dropout, is becoming increasingly urgent.

## Data availability statement

The original contributions presented in the study are included in the article/supplementary material, further inquiries can be directed to the corresponding author/s.

## Author contributions

DY and KW contributed to the conception and design of the article and interpreting the relevant literature and drafted the manuscript and revised it substantively. PC, HW, and RH contributed to the interpretation of data, and revised it critically for important intellectual content. All authors read and approved the final manuscript.

## Conflict of interest

The authors declare that the research was conducted in the absence of any commercial or financial relationships that could be construed as a potential conflict of interest.

## Publisher's note

All claims expressed in this article are solely those of the authors and do not necessarily represent those of their affiliated organizations, or those of the publisher, the editors and the reviewers. Any product that may be evaluated in this article, or claim that may be made by its manufacturer, is not guaranteed or endorsed by the publisher.
